# Microsurgical treatment of carotid-ophthalmic aneurysm associated with multiple anterior and posterior circulation aneurysms: A case report

**DOI:** 10.1097/MD.0000000000006672

**Published:** 2017-04-21

**Authors:** Jiantao Wang, Zhisheng Kan, Shuo Wang

**Affiliations:** a Department of Neurosurgery, Beijing Tiantan Hospital, Capital Medical University, Beijing, China; b Department of Neurosurgery, Beijing Anzhen Hospital, Capital Medical University, Beijing, China.

**Keywords:** carotid-ophthalmic artery aneurysm, classification, clipping, multiple

## Abstract

**Background:**

The clipping of multiple intracranial aneurysms in 1 stage is uncommon. In this case, we report clipping of an ophthalmic aneurysm associated with multiple anterior and posterior circulation aneurysms via the Dolenc approach.

**Methods:**

The main symptoms of the patient are headache, along with nausea and vomiting. The patient's arteriogram revealed a wide-necked aneurysm of the right ophthalmic artery, an irregular aneurysm of the anterior communicating artery, and a basilar artery aneurysm. The surgical intervention for these aneurysms is a challenge because of the complex anatomical relationship with the surrounding structures. The 3 aneurysms, which were not amenable to a single intervention, were successfully clipped in 1 incision.

**Results:**

After surgery, the patient reported feeling well. One year after surgery, the patient had no SAH recurrence.

**Conclusions:**

Occasionally, surgical treatment was used even for aneurysms of the carotid-ophthalmic artery with aneurysms of anterior communicating artery and basilar artery, which are contraindicated for interventional therapy.

## 1 Introduction

Ophthalmic internal carotid artery (ICA) aneurysms are a challenging subset of intracranial aneurysms. The ophthalmic (C6) segment extends from the distal dural ring to the origin of the posterior communicating artery.^[1]^ The segment is known as the carotid-ophthalmic segment^[2]^ and the paraclinoid segment.^[3]^ Internal carotid artery (ICA)-ophthalmic artery aneurysms constitute 0.3% to 1% of intracranial aneurysms and 0.9% to 6.5% of aneurysms of the ICA.^[4]^ They represent a surgical challenge because of the anatomical complexity of the paraclinoid region, and proximity to the optic apparatus, as well as partial intracavernous extension in a few patients.^[5]^ We report the surgical clipping of carotid-ophthalmic aneurysm in patients with multiple anterior and posterior circulation aneurysms intracranially.

## 2 Case report

A 47-year-old woman with unremarkable medical history presented with sudden headache, along with nausea and vomiting. Physical examination revealed a stiff neck. Visual acuity and field were within normal limits, with Hunt & Hess grade II.

**Figure 1:**
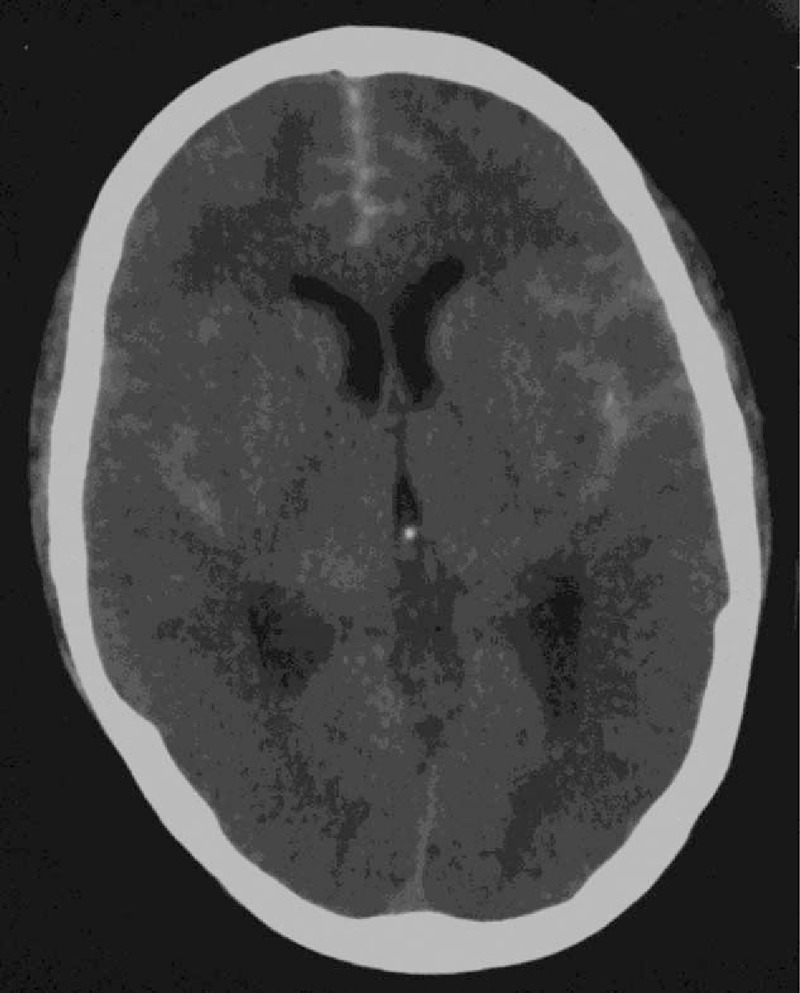
CT demonstrating subarachnoid hemorrhage. CT = computed tomography.

**Figure 2:**
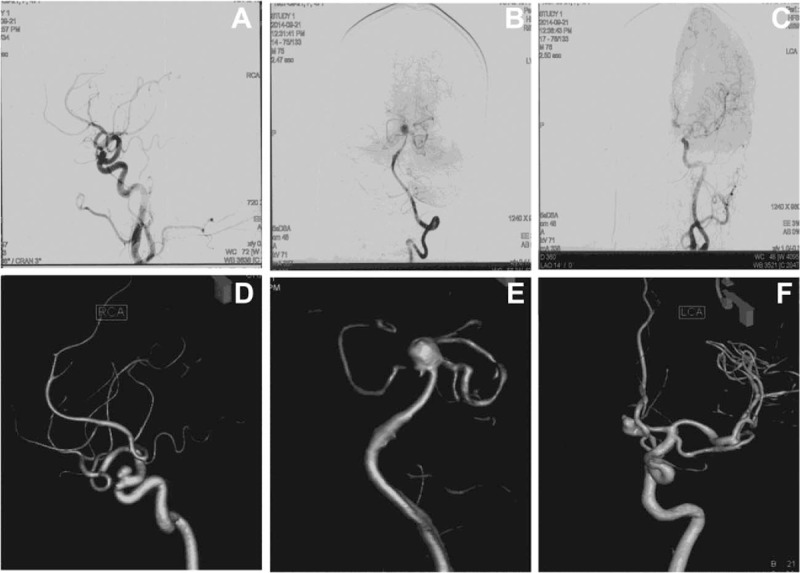
(A, D) Right carotid angiography demonstrated a carotid-ophthalmic artery aneurysm with upper projection. (B, E) Left vertebral angiography demonstrated a basilar artery aneurysm. (C, F)Left carotid angiography demonstrated an anterior communicating artery aneurysm.

**Figure 3:**
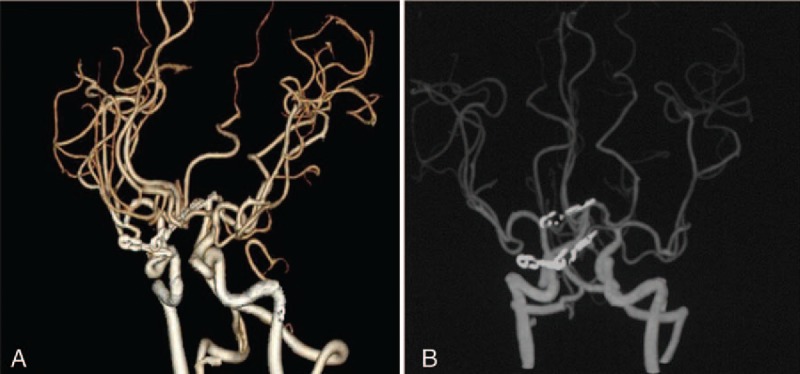
Postoperative CTA showing clipped aneurysms. CTA = computed tomographic angiography.

The CT scan showed subarachnoid hemorrhage (Fig. 1). Digital subtraction angiography (DSA) with 3-dimensional reconstruction revealed a 6-mm wide-necked aneurysm of the right ophthalmic artery projecting superomedially, a 8-mm saccular aneurysm on the top of basilar artery and an irregular 5-mm aneurysm of anterior communicating artery projecting anterosuperiorly, which caused the hemorrhage (Fig. 2). The aneurysms were successfully obliterated with microsurgical clipping using a single craniotomy. Postoperative computed tomographic angiography (CTA) demonstrated complete disappearance of all the aneurysms (Fig. 3), and the patient was discharged after 14 days without any neurological deficits. The patient is alive and healthy without any neurological deficits, 1 year after surgery.

**Figure 4:**
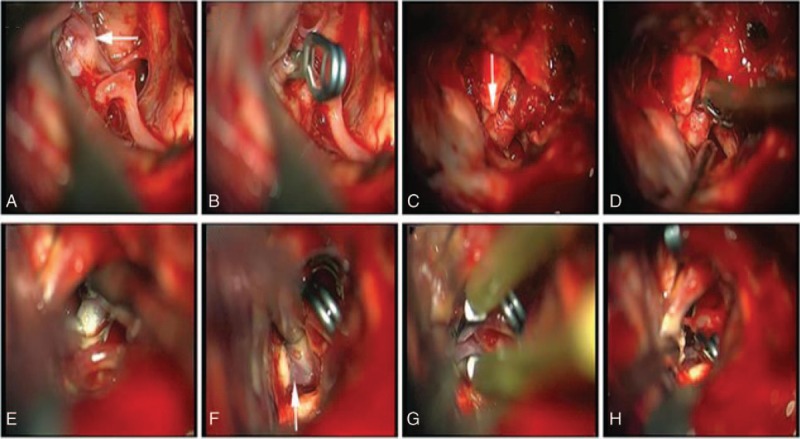
Intraoperative view of (A) anterior communicating artery aneurysm (ACOA); (B) clip across the ACOA neck; (C) ophthalmic artery aneurysm (OAA); (D) clip across the OAA neck; (E) blocking of temporary proximal basilar aneurysm (BA); (F) BA; (G) BA size was reduced using low-power electrocautery; (H) clip across the BA neck. ACOA = anterior communicating artery aneurysm, BA = basilar aneurysm, OAA = ophthalmic artery aneurysm.

The case is unique in that the 3 aneurysms were successfully clipped in 1 incision using extended pterional craniotomy with a temporal extension, to expose the cervical carotid arteries initially. After the craniotomy flap was established, the sphenoid wing was drilled laterally to its medial extension until the anterior clinoid process was reached. The anterior clinoid process was exposed and removed extradurally. During exposure and surgical clipping of the aneurysm, a sharp dissection was used to open the arachnoid of the lamina terminalis cistern and the arachnoid between the optic nerves and gyrus rectus. A straight clip was applied across the neck of the anterior communicating artery aneurysm (Fig. 4A and B). The microtechnique was continued by extending the dissection laterally to the right internal carotid artery. The paraclinoidal aneurysm was dissected and clearly visualized (Fig. 4C). The aneurysm originating in the dorsal surface of the C6 segment and was close to the ophthalmic artery origin. The subtype Ia^[6]^ was close to the ophthalmic artery origin, and a straight aneurysm clip was applied across the neck of the aneurysm (Fig. 4D). Along the direction of the posterior cerebral artery, the basilar trunk was directly exposed in the interpeduncular cistern. A saccular aneurysm with a wide and dysmorphic base was detected at the top of the basilar artery. After blocking the temporary proximal aneurysm, the size of the aneurysm was reduced using low-power electrocautery (Fig. 4E–G). A straight aneurysm clip replaced the temporary clip across the neck of the aneurysm (Fig. 4H).

## 3 Discussion

We report a patient with multiple aneurysms located in the carotid-ophthalmic artery, the anterior communicating artery and the basilar artery. We used a single craniotomy to successfully clip all the aneurysms, which were refractory to single intervention.

Based on DSA information, ophthalmic segment aneurysms are classified into 4 groups as reported by Barami et al.^[6]^ Types Ia and Ib originate on the dorsal surface of C6. Type Ia is related to the ophthalmic artery. Type Ib aneurysms are sessile, without any branch points. From a surgical perspective, the type I aneurysms are easiest to treat, with limited correlation with superior hypophysial vessels or other strategically important vessels. Frequently, only the optic nerve and the OphA artery must be negotiated. Surgery is the first-line therapy for paraclinoid aneurysms (Type I), unless the neck of the aneurysm is heavily calcified or contraindications exist.^[7]^ The ophthalmic segment aneurysm is classified under the type I category.

Paraclinoid aneurysms represent one of the most appropriate targets for endovascular intervention. However, endovascular treatment has a lower success rate for total occlusion.^[8,9]^ In addition, it is associated with recurrence, especially, of lesions incompletely occluded initially.^[10,11]^ Microsurgery remains the primary treatment for ICA aneurysms of the paraclinoid segment, resulting in a higher rate of long-term success.^[11]^ Occasionally, the combination of surgical and endovascular approaches is an effective strategy.^[12]^ Anticoagulation after intervention leads to ruptured aneurysm, and patients with multiple aneurysms may be contraindicated for interventional therapy. In our patient with ophthalmic segment aneurysm, young age and good health aneurysm location and the wide neck of the other aneurysms are indications for surgical clipping.

The contraindications for interventional therapy of aneurysms are discussed below.

1. The type 1 or superior projecting paraclinoid aneurysms showing instability of the microcatheter are inappropriate for intervention. By contrast, aneurysms projecting inferiorly (ventral paraclinoid aneurysms), in which the location of aneurysms within the concavity of the curve formed by the carotid siphon facilitate catheterization.^[7]^
2. Anticoagulation leads to rupture in patients with multiple aneurysms.
3. Other factors, such as aneurysm shape and neck size, as well as the wishes of patient's family affect the treatment. Wide-necked basilar artery aneurysms are difficult to treat using endovascular therapy. Therefore, a temporary blockage of proximal aneurysm was used to reduce the size using low-power electrocautery followed by straight clipping across the neck of the aneurysm.

The optimal therapy for multiple intracranial aneurysms remains unclear. Elective surgery is recommended for unruptured intracranial aneurysms. The risk of rupture was high in our patient with SAH. When surgery is indicated for ruptured aneurysm, the additional effort to clip the other unruptured aneurysms is minimal, obviating the need for a second craniotomy. Although surgical indications for ophthalmic segment aneurysms are minimized with the success of endovascular techniques, multiple aneurysms are still a challenge due to their different locations. However, long-term follow-up data show higher rates of recurrence and re-treatment with endovascular intervention, and surgical clipping continues to be strongly preferred.^[13]^ Although multiple aneurysms are difficult to treat with surgical clamping, the use of appropriate surgical methods, adequate exposure, and clipping facilitate successful management. We used the extradural approach adopted by Dolenc^[14]^ for paraclinoid aneurysms and basilar tip,^[15]^ to treat the aneurysms of the anterior communicating artery, the basilar artery, and the paraclinoid segment.

The anterior clinoid process (ACP) interferes with clipping. It is necessary to remove the ACP followed by optic canal unroofing to expose the ophthalmic segment aneurysm. The ACP resection can be performed intradurally or extradurally. The proponents of extradural clinoidectomy maintain that the dural layer protects the brain and cortical vessels during the drilling, and prevents bone dust and bleeding into the subarachnoid space.^[1]^ By contrast, intradural clinoidectomy provides a clear view of the ACP, ICA, and optic nerve, which are protected during clinoidectomy. We treated our case with extradural clinoidectomy.

Our experience suggests that surgical treatment is superior to endovascular treatment. Surgical clipping releases optic nerve compression and completely occludes the aneurysm neck. It provides durable repair, without the need for antiplatelet agents in the setting of acute aneurysm rupture.

As a cost-effective, noninvasive modality, CTA is a promising alternative to DSA for initial and long-term evaluation of residual cerebral aneurysms (RA), although DSA remains the gold standard.^[16]^ Therefore, we used CTA as a means of postoperative assessment of RA. By implementing multidetector CTA technology in experienced centers, the sensitivity and specificity of CTA may approach that of traditional DSA for detecting RA.^[16]^

## 4 Conclusion

Single craniotomy was successfully used to clip ophthalmic segment aneurysms associated with other aneurysms. Despite a narrow range of indications for endovascular interventions in patients with ophthalmic segment aneurysms, management of multiple aneurysms is still a challenge due to their different locations. The successful outcome reported here defines the feasibility but not the efficacy of endovascular approach. The procedure requires extreme prudence along with adequate experience and skills when used as a possible alternative to other well-established techniques.
